# Methods to Assess the Protective Efficacy of Emollients against Climatic and Chemical Aggressors

**DOI:** 10.1155/2012/864734

**Published:** 2012-08-16

**Authors:** Romain Roure, Marion Lanctin, Virginie Nollent, Christiane Bertin

**Affiliations:** R & D Scientific Affairs, Johnson & Johnson Santé Beauté France, 1 rue Camille Desmoulins, Issy-les-Moulineaux, France

## Abstract

Exposure to harsh environmental conditions, such as cold and dry climate and chemicals can have an abrasive effect on skin. Skin care products containing ingredients that avert these noxious effects by reinforcement of the barrier function can be tested using *in vivo* models. The objective is to use in vivo models to assess the efficacy of emollients in protecting skin against climatic and chemical insults. A first model used a stream of cooled air to mimic cold wind. A second used sodium lauryl sulfate (SLS) under patch as chemical aggressor. In the model with simulated wind exposure, the untreated exposed area had a significant decrease in hydration. In contrast, application of an emollient caused a significant increase in hydration that was maintained after wind exposure. In the second model with SLS exposure, application of a barrier cream before SLS patch significantly reduced the dehydrating effect of SLS with a significant difference in variation between both areas. Application of the cream reduced TEWL, indicative of a physical reinforcement of the skin barrier. The two presented test methods, done under standardized conditions, can be used for evaluation of protective effect of emollient, by reinforcing the barrier function against experimentally induced skin dehydration.

## 1. Introduction

The skin is the outermost barrier that protects the human body from physical, chemical, and microbial insults and prevents the uncontrolled loss of water among other substances. The epidermal barrier function of the skin resides in the stratum corneum (SC) and is linked to the protein-enriched corneocyte layers and the intercellular membrane lipids mostly composed of ceramides, cholesterol, and free fatty acids [1, 2]. Corneocytes are rapidly and continually replaced to maintain skin integrity and to repair damage. 

Exposure to external factors can damage this protective function. Much studied is the cumulative damage of sun exposure as it accounts to a great extent for the permanent changes in skin physiology and morphology over time [3]. Other environmental aggressors that significantly impact on skin properties and may cause acute or chronic damage of the skin barrier include climatic conditions (e.g., wind, low temperatures, low humidity) and chemicals in frequent contact with the skin (e.g., soaps and detergents) [4, 5]. Exposure to such aggressors can reduce the hydration status of the epidermis and compromise the skin barrier function. Skin dryness reflects an abnormal desquamation process, where corneocytes are shed as visible scales, causing the cosmetically unattractive rough texture associated with dry skin and provoking discomfort and itchiness. Compromised skin barrier shows visible irritation (redness) or even inflammation. Moreover, dry and barrier impaired skin favors penetration of microorganisms, allergens, and other irritants. Dryness and impaired barrier function are also symptoms of inflammatory skin diseases such as atopic dermatitis (AD) [6], the etiology of which is determined by a range of factors, including genetic, immunological, environmental factors (such as cold climate), and chemical and mechanical irritants [7, 8].

There is thus a need to protect both healthy and sensitive skin from environmental and chemical aggressors and to preserve or restore its integrity. Cosmetic products containing emollients (also referred to as moisturizers) are specifically formulated to soften and soothe dry skin and to reduce itching sensation and irritation signs. Emollients are delivered in the form of creams, ointments, gels, pastes, or liquid preparations [9]. They increase the moisture content of the SC by providing an occlusive oily film on the skin surface to reduce transepidermal water loss (TEWL), which is the normal movement of water through the SC, and by serving as humectants, that is, binding water and thus increasing the water holding capacity in the SC. Emollients thus prevent and alleviate skin dryness by increasing skin hydration and reducing TEWL and promote recovery of the damaged skin barrier, including that observed in atopic skin. Formulations contain a combination of ingredients, including emollient lipids (e.g., mineral oils, waxes, fatty acids, and glycerides), humectants (e.g., alpha-hydroxy acids, urea, and glycerin), emulsifiers, and antipruritics (e.g., glycine), as well as inactive components. 

A large number of emollient formulations exist, more or less effective in their proposed functions [10, 11]. Standardized, controlled testing conditions are thus crucial in the development of adequately formulated products. The goal of this study was to implement models that allow assessing the efficacy of emollients in protecting skin against climatic and chemical insults under standardized conditions *in vivo*. We designed two models, one in which the effect of cold and dry wind was mimicked by exposure to a continuous stream of cooled air and the second, with sodium lauryl sulfate (SLS) under patch used as chemical aggressor. 

## 2. Materials and Methods

For the simulation of cold and dry wind, compressed air was led through a temperature controlled rubber tube (stabilized at 13 ± 2°) and the airflow maintained constant was blown onto the skin for 15 minutes. The hydration level of the SC was assessed by determination of capacitance using corneometry (Corneometer, Courage + Khazaka electronic GmbH) and results were expressed in arbitrary units [12]).

To simulate the exposure to chemical aggressors, a patch consisting of 1% SLS solution was applied to the skin for 3 hours. The hydration level of the SC was assessed by determination of capacitance using corneometry (Corneometer, Courage + Khazaka electronic GmbH) and results were expressed in arbitrary units [12]). Moreover, the transepidermal water loss (TEWL) was assessed using a Tewameter (Courage + Khazaka electronic GmbH) and results were reported in g/m^2^/h [13].

Two studies were conducted to assess the potential of emollient containing skin care products to protect skin from exposure to cold, dry air and to SLS under controlled laboratory conditions. Subjects were acclimated to testing conditions for 15 minutes at a temperature of 20 ± 2°C and a relative humidity of 50 ± 5% before measurements. All subjects gave their written informed consent prior to study enrolment. The studies were conducted in accordance with the Declaration of Helsinki [14]. All adverse events, whether considered product related or not, were reported during these studies. 

### 2.1. Simulated Exposure to Cold and Dry Wind

Twelve healthy Caucasian women of normal skin type and aged between 53 and 70 years participated in this study. The test product was a lotion containing glycerin and glycine soja as emollients. A fixed quantity (2 *μ*l/cm^2^) of the test product was applied to the inner forearm on two different test areas of 16 cm^2^ size. Three test areas were assessed in each subject (1) treated area (with test product), without wind exposure; (2) untreated area, with wind exposure; (3) treated area, with wind exposure. Measurements were taken at the following time points (1) treated area, without wind exposure: before test product application (*T*_0_), 1 hour after application (*T*_after  lotion_), and 5 minutes after a 15 minutes resting period (*T*_after  resting_, replacing wind exposure); (2) untreated area, with wind exposure: before wind exposure (*T*_0_) and 5 minutes after 15 minutes wind exposure (*T*_after  wind_); (3) treated area, with wind exposure: before test product application (*T*_0_), 1 hour after application (*T*_after  lotion_), and 5 minutes after 15 minutes wind exposure (*T*_after  wind_). Three consecutive measurements were taken at each test area and at each time point. 

Statistical analysis included the calculation of mean values and standard deviation (SD) at all time points, as well as percentage of variation (%) relative to *T*_0_ using Microsoft Excel 2000. The results were compared with the paired bilateral Student's *t*-test and the level of statistical significance was set at *P* ≤ 0.05. Variance analysis was performed to compare the variation between *T*_after  wind/lotion_ to *T*_0_ between the three assessed areas using the Fisher's least significant difference (LSD) test (StatGraphics Plus 5.1 software).

### 2.2. Exposure to a Chemical Aggressor (Sodium Lauryl Sulfate (SLS))

Fourteen healthy Caucasian women of normal skin type and aged between 25 to 68 years participated in this study. The test product was a barrier cream (nappy cream) containing glycerin, sorbitol, and butylene glycol as emollients associated to zinc oxide. A fixed quantity (2 *μ*l/cm^2^) of the test product was applied to the inner forearm at two different test areas of 16 cm^2^ size. SLS application consisted of a 1% SLS solution applied to the skin under a semiocclusive patch for 3 hours. Three test areas were assessed (1) treated area (with test product), without SLS exposure; (2) untreated skin area, with SLS exposure; (3) treated area, with SLS exposure. At the test area without SLS exposure an empty patch was applied. Where applicable, the test product was applied before the SLS patch. Three repetitive measurements with Corneometer were taken at each test areas and at each time points. Two consecutive measurements with Tewameter were carried out before (*T*_0_) and one hour after product application (*T*_1 h_) on the untreated control area (without product) and the treated area.

Mean values and SD at all time points and variation (%) at *T*_30 min⁡_ relative to the untreated area and *T*_0_ were calculated. Data were compared using the paired Student's *t*-test for normal distribution and the Wilcoxon signed rank test for not normal distribution. Statistical significance was defined as *P* ≤ 0.05. Matlab was used for statistical analysis. Variance analysis was performed to compare the variation between *T*_30 min⁡_ and *T*_0_ between the different test areas using ANOVA.

## 3. Results

### 3.1. Exposure to Cold and Dry Wind

**Figure 1:**
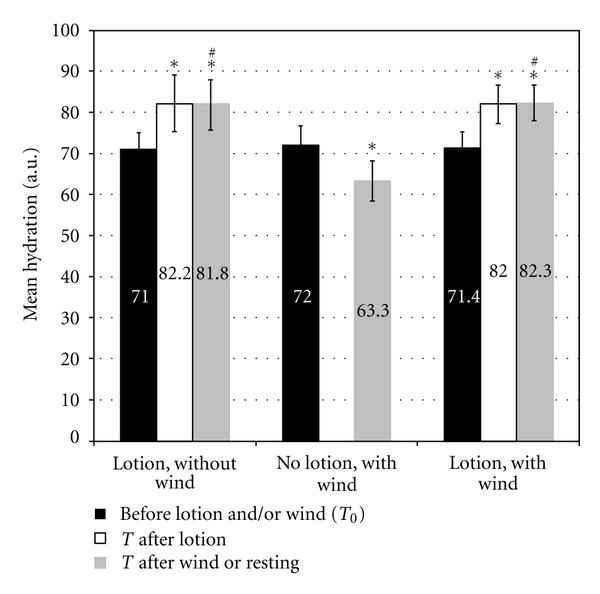
Hydration after wind exposure with and without an emollient lotion. *indicates a statistically significant difference compared to *T*_0_ (*P* < 0.001). ^#^indicates a statistically significant difference in the variation between *T*_after  wind/resting_ and *T*_0_ between the two lotion-treated test areas (with and without wind) and the untreated area (no lotion, with wind) (*P* < 0.05, variance analysis).

The results of the twelve subjects participating in this study were analyzed. The results of the hydration assessment at the three different test areas are shown in Figure 1. At the lotion treated area, where wind exposure was substituted by a resting period, there was a significant increase in hydration at *T*_after  lotion_ with a variation of 15.8% (*P* < 0.001) compared to *T*_0_. At *T*_after  resting_, hydration remained at a similar level compared to *T*_after  lotion_ (variation 15.1% compared to *T*_0_, *P* < 0.001). At the untreated area, a significant decrease in skin hydration was noted after wind exposure at *T*_after  wind_ with a variation of −12.1% (*P* < 0.001) compared to *T*_0_. In contrast, hydration measured at the treated test area exposed to wind was significantly increased at *T*_after  lotion_ and remained at a similar level at *T*_after  wind_. The variations compared to *T*_0_ were 14.8% and 15.2% (*P* < 0.001 for both time points), respectively, at these two time points and thus similar to the changes observed at the treated area, without wind. The variance analysis showed that the variation between *T*_after  wind/rest_ and *T*_0_ was significantly different at the treated, with wind area as well as at the treated, without wind area compared to the untreated, with wind area (*P* < 0.05).

### 3.2. Exposure to SLS under Patch

**Figure 2:**
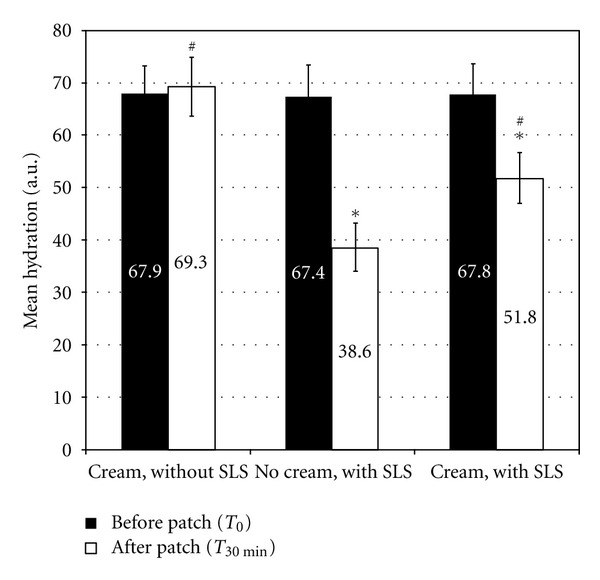
Hydration after SLS exposure with and without an emollient cream. *indicates a statistically significant difference compared to before cream application (*P* < 0.001). ^#^indicates a statistically significant difference in the variation between *T*_30 min⁡_ and *T*_0_ between the two cream-treated test areas (with and without SLS) and the untreated test area (no cream, with SLS) (*P* < 0.05, variance analysis).

The results of the 14 subjects participating in this study were analyzed. Hydration assessment showed the following results for the three test areas (Figure 2): test product application without subsequent SLS exposure led to a nonsignificant increase in hydration with a variation of 2.1% at *T*_30 min⁡_ compared to *T*_0_. When the test area was exposed to SLS without previous test product application, there was a significant decrease in skin hydration at *T*_30 min⁡_ with a variation of −41.7% compared to *T*_0_ (*P* < 0.001). Application of the test product before SLS exposure resulted in a significant decrease in hydration with a variation of −23.6% compared to *T*_0_ (*P* < 0.001). According to the variance analysis, the change between *T*_30 min⁡_ and *T*_0_ was significantly smaller at the cream treated area with SLS exposure compared to the area not treated before SLS exposure (*P* < 0.05).

**Figure 3:**
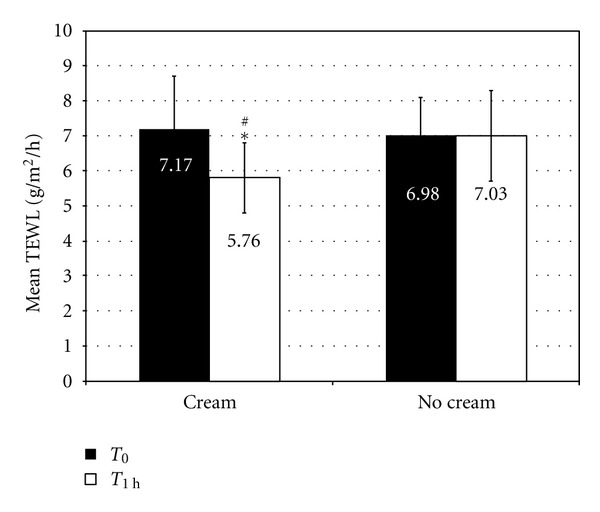
TEWL with and without emollient cream.*indicates a statistically significant difference between *T*_1 h_ and *T*_0_ (*P* = 0.002). ^#^indicates a statistically significant difference in the variation between *T*_1 h_ and *T*_0_ between the two areas.

The TEWL measurements showed a significant decrease at *T*_1 h_ compared to *T*_0_ when emollient was applied (variation −19.6%, *P* = 0.002). Moreover, the statistical comparison between areas showed a significant difference (*P* = 0.002) (Figure 3). 

There were no adverse events reported in either of the two studies.

## 4. Discussion

Emollient use in the form of bath additives, creams, lotions, or ointments is recommended to relief dry and itchy skin conditions and as adjuvant therapy in the management of skin barrier disorders such as AD [6, 15–17]. It was observed that the beneficial effect of emollients for skin barrier restoration allows for a significant reduction of the use of high-potency topical corticosteroid consumption to diminish disease severity in AD afflicted infants [18]. 

The surfactant SLS is a common ingredient in personal care products and is used as a model substance to experimentally elicit skin barrier damage. Depending on the concentration and exposure conditions, SLS can provoke skin dryness, roughness, tightness, erythema, and inflammation. This is related to the potential of surfactants to denature proteins in the SC, solubilize intercellular skin lipids, increase the skin pH, and increase TEWL [19]. Repeated exposure to surfactants, as is the case in frequent hand washing, causes in many individuals irritant contact dermatitis, characterized by inflammation and pruritic lesions of the skin. 

Rough climatic conditions also impact on skin integrity, as seen in cold and dry winter months. Exposure of skin to a dry environment reduces the SC water content and induces changes in the skin surface texture [20]. In addition, wind removes water vapor from the skin causing redness and chafing [21]. These changes are usually reversible and skin hydration tends to improve during more humid summer months [22]. 

Some recent controlled studies have demonstrated the beneficial effect of emollients on skin dryness and irritation associated with exposure to dry and cold climatic conditions and to the irritation potential of repeated hand washing. For example, regular application of an emollient containing body-wash reduced the signs of xerosis associated with dry winter skin compared to a regular bar cleanser [23]. Likewise, regular application of some (but not all) tested moisturizing creams reduced the risk of skin irritation linked to repeated hand washing with soap in healthy skin [10]. In another study, repeated hand immersion into an SLS solution caused barrier dysfunction with increased TEWL and reduced skin hydration, which was prevented when the skin was preventively treated with a moisturizer [24].

The environmental and chemical insults modeled in our studies were rather mild in nature and did not induce skin irritation beyond skin dehydration. In an approach similar to ours, Cheng et al. evaluated the effect of two cosmetic products on skin water content and TEWL under simulated wind exposure [25]. However, the conditions chosen in their study were not harsh enough to induce a significant change in these parameters compared to unexposed skin. 

Our model with experimentally induced skin dehydration with cold and dry wind permitted us to distinguish the effect of the emollient containing test product from no treatment. At the area treated with lotion and exposed to wind there was no significant decrease in hydration in contrast to the unprotected area, demonstrating a protective effect of the lotion. Moreover, many tests using this model have been performed on different emollient products since several years. In each test, we observed that, without product, the dehydration is at the same level and the application of product allows protecting the skin from the drying out. However, TEWL measurements were not performed. Indeed, when this model was developed, it has been demonstrated that hydration measurements seemed to be the most relevant measurements for this model, in our experimental conditions. In a long-term study, Black et al. have showed that the TEWL only significantly changed in summer. Decreases in lipids (ceramides and cholesterol) and in hydration (electrical conductance) of the *Stratum corneum* were the main changes observed in winter situation [22]. Many tests using this model have been performed on different emollient products since several years. In each test, we observed that, without product, the dehydration is at the same level and the application of product allows protecting the skin from the drying out.

In the second model, skin dehydration was induced by subirritant exposure to 1% SLS under patch. We found that skin hydration was significantly reduced when no emollient containing test product was applied prior to SLS exposure. In the presence of the cream the loss of skin hydration was significantly smaller, indicating a protective effect of the cream. Besides, the skin barrier function was significantly reinforced in unexposed skin in the presence of the cream, as indicated by reduced TEWL.

Emollients are beneficial for the reinforcement of both normal and sensitive skin. Irritant contact dermatitis is of particular relevance in infants, as they are prone to develop irritant contact dermatitis in the diapered area due to prolonged exposure to urine, feces, high skin pH, and chemical irritants from the diaper. Alkaline conditions activate intestinal enzymes, which together with excessive hydration and friction leads to skin barrier breakdown and irritation [26]. The application of a water repellent emollient is recommended in the management of diaper dermatitis [27].

## 5. Conclusions

The two devised methods mimicking cold and dry wind and surfactants insults, done under standardized conditions, can be used for evaluation of protective effect of emollient. The protection brought by the emollients can then be assimilated to a reinforcement of the barrier function.
